# Thermocycling: enhancing efficiency of anesthetic reflectors through cyclic heating and cooling—finding the optimal temperature in a bench study

**DOI:** 10.1186/s40635-026-00905-5

**Published:** 2026-04-28

**Authors:** Frederic Albrecht, Kathrin Scheffler, David Kellner, Ilayda Camyurdu, Thomas Volk, Andreas Meiser

**Affiliations:** 1 Department of Anesthesiology, Intensive Care and Pain Therapy, Saarland University Medical Center, Homburg, Germany; 2 Faculty of Medicine, Saarland University, Homburg, Germany; 3 Outcomes Research Consortium®, Houston, TX USA

**Keywords:** Thermocycling, Volatile anesthetics, Sevoflurane, Isoflurane, Green anesthesia, Mechanical ventilation, Sedaconda ACD-S

## Abstract

**Background:**

Volatile anesthetics are widely used for sedation in intensive care. Being potent greenhouse gases, their efficient utilization is imperative. We investigated whether the efficiency of anesthetic reflection devices, such as Sedaconda ACD-S (ACD), can be improved by thermocycling, i.e., cooling the reflector during expiration and warming it during inspiration, and aimed to identify the optimal temperatures required.

**Methods:**

A test lung connected to the ACD was ventilated under body temperature pressure saturated and normocapnic conditions. Isoflurane and sevoflurane were infused at rates of 0.5, 1, 2, and 5 mL/h, with sevoflurane additionally administered at 10 mL/h. For thermocycling, inspired air was heated to 37 °C by an active humidifier without water. Cooling in steps of 26, 21, 16, 11, 6, and 1 °C was achieved by passing air from the test lung through a freezer before reaching the ACD.

**Results:**

Thermocycling significantly increased concentrations of isoflurane and sevoflurane in the test lung compared with control conditions. Cooling of the expired air led to substantial increases down to a cooling temperature of 16 °C; below 16 °C, further increases in concentrations were much smaller. Interpolation of our data shows that at clinically used concentrations (isoflurane: 0.4–0.6 Vol%; sevoflurane: 0.9–1.1 Vol%), consumption could be reduced by 70% (isoflurane from 2.46 to 0.74 mL/h) and 72% (sevoflurane from 5.73 to 1.60 mL/h). Reflection efficiencies—the ratio of re-inspired from exhaled anesthetic molecules in one breath—increased from around 70% to 90%.

**Conclusions:**

Thermocycling significantly enhances the efficiency of volatile anesthetic reflection, offering a promising strategy to reduce the impact of intensive care sedation on climate change.

## Background

Critically ill patients in Intensive Care Units (ICU) often require continuous sedation to tolerate endotracheal intubation, mechanical ventilation, and other invasive procedures. Recently, volatile anesthetics have emerged as alternative agents for sedation in the ICU [1–3].

Nevertheless, the ecological impact of volatile anesthetics as greenhouse gases is a growing concern, given their considerable global warming potential [4]. Isoflurane and sevoflurane are currently regulated under the European Union’s framework on fluorinated greenhouse gases [5]. Therefore, efficient utilisation of these agents is imperative to mitigate their impact on climate change.

Unlike operating rooms, which routinely use integrated vaporizers, volatile agents in the ICU are typically administered via devices positioned between the Y-piece of the ventilator circuit and the patient’s endotracheal tube [6, 7]. These devices partially recycle exhaled anesthetic through adsorption onto an internal activated carbon matrix during expiration and subsequently release it during inspiration in a process called anesthetic reflection [8, 9].

Temperature has been identified as a critical factor affecting the adsorption–desorption dynamics, with lower temperatures enhancing adsorption to the activated carbon reflector, while higher temperatures facilitate desorption. Building on this observation, thermocycling—cooling the reflector during expiration and warming it during inspiration—has been shown to improve isoflurane reflection [10].

This bench study evaluated the impact of thermocycling on the efficiency of the reflector inside Sedaconda ACD-S (Sedana Medical AB, Danderyd, Sweden) under body temperature and pressure saturated (BTPS) plus normocapnic conditions in a test lung, specifically analyzing isoflurane and sevoflurane. We hypothesized that cyclic temperature modulation would enhance the adsorption–desorption cycle, thereby reducing anesthetic consumption compared to unmodulated controls. In addition, the study explored different intensities of thermocycling, aiming to identify the optimal range for enhancement of anesthetic reflection.

## Methods

### Experimental setup

**Fig. 1:**
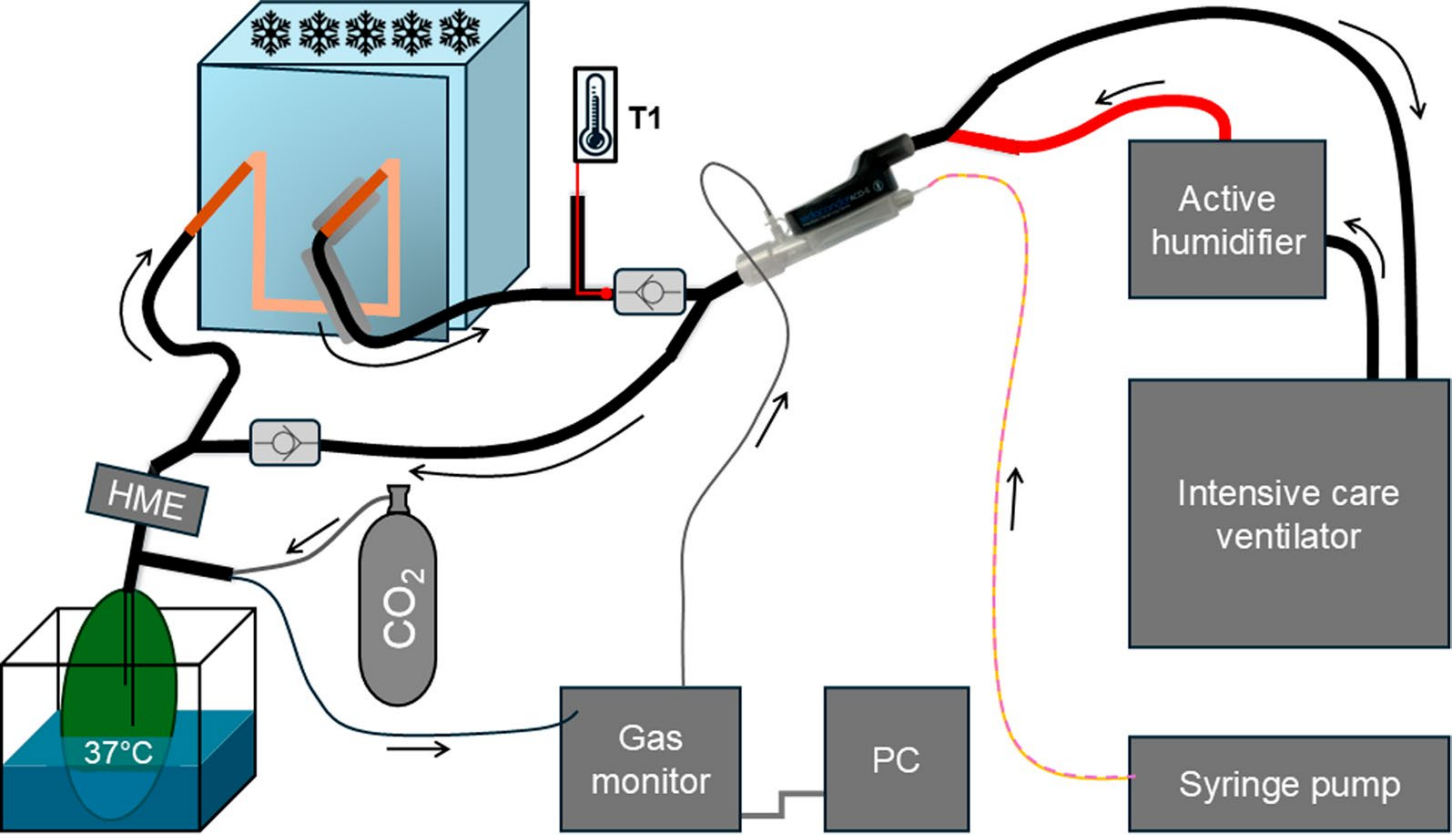
Experimental setup, T1: thermometer; HME: heat and moisture exchanger

A comparable experimental setup has previously been described [9, 10]. In the present setup (Fig. 1), a freezer was integrated to achieve different temperature steps through adjustable freezer settings.

An intensive care ventilator (Evita 4, Draeger Medical, Luebeck, Germany) was connected to a test lung (3L Chloroprene breathing bag, Intersurgical Ltd, Berkshire, UK) via a Sedaconda™ ACD-S. The anesthetic (isoflurane or sevoflurane) was delivered into the ACD using a syringe pump (Perfusor® fm, B.Braun, Melsungen, Germany). The inspiratory limb from the ventilator was heated to 37 °C using an active humidifier (MR850, Fisher & Paykel Healthcare Limited, Auckland, New Zealand), controlled by a temperature probe placed close to the Y-piece. This active humidifier was used without water, ensuring that the gas reaching the ACD was dry and warm.

Before reaching the test lung, the gas passes through a tube extension containing a directional valve (Intersurgical Ltd, Berkshire, UK), a Y-piece, and a heat and moisture exchanger (HME, Humid-Vent® Filter Compact, Teleflex Medical, Fellbach, Germany).

The test lung was positioned in an aquarium (Fluval glass aquarium, Hagen Deutschland, Holm, Germany), filled with 20 L of distilled water continuously heated to 42 °C using a heat circulation pump with a heater and thermostat (Kryothermostat WK 5, Colora Messtechnik, Lorch, Germany), targeting 37 °C inside the test lung. This temperature was continuously monitored with a temperature probe (Terracheck, TFA Dostmann, Wertheim-Reicholzheim, Germany), placed centrally inside the test lung. The probe was introduced via a T-piece (Dahlhausen, Cologne, Germany), one side of which was sealed with hot glue, and mounted onto the test lung. In addition, 100 mL of distilled water was filled into the test lung to achieve a relative humidity above 95%, verified after each experiment using a hygrometer (Testo 610, Testo SE & Co. KGaA, Titisee-Neustadt, Germany). These conditions correspond to BTPS.

CO₂ was supplied to the test lung via a gas line (sample line, Dräger Medical, Lübeck, Germany), while another gas line transferred sample gas to a gas monitor (Vamos®, Dräger Medical), where real-time CO₂ and anesthetic concentrations were measured and recorded on a computer. Both lines were entering the lung through the T-piece. The sample gas was then returned to the ACD.

The expiratory limb included tube elongations and a copper tube passing through a freezer (AY7107, BD-40, ARTE Living, Hilpoltstein, Germany) to cool the exhaled air. Various approaches were utilized to modulate cooling in steps of 5 °C. The freezer was set to one of the five temperature levels depending on the targeted degree of cooling. Additional regulation was achieved by adjusting the freezer door to be slightly open or fully closed. Insulation (15mm CLIMAFLEX®, NMC, Eynatten, Belgium) was applied to the expiratory limb segment exiting the freezer. Furthermore, the copper tube was inserted progressively deeper into the freezer to enhance cooling. In the final experimental condition, a bucket containing antifreeze liquid (radiator antifreeze − 35 °C, Walter Schmidt Chemie, Berlin, Germany) was placed around the copper tube to achieve the lowest exhaled air temperature. The temperature of the cooled gas was monitored at the end of the expiratory limb, immediately before entering a directional valve connected to a Y-piece and the ACD. After the adjustments were performed to achieve the target temperature, the stability of the temperature setting was first verified. The experiment began if the temperature stayed within 0.3 °C of the target for at least 30 min.

The control setup was built up to simulate the configuration used in clinical practice for critically ill patients as closely as possible; therefore, additional thermocycling components were omitted. These control measurements were performed using a shorter tubing system without active heating and cooling. BTPS and normocapnia were always maintained.

The test lung was ventilated using volume-controlled ventilation with the following settings: tidal volume 500 mL, respiratory rate 12 breaths/min, constant inspiratory flow 25 L/min, inspiration time 1.6 s, inspiratory oxygen fraction 0.21, and positive end-expiratory pressure (PEEP) 5 mbar.

Anesthetic concentrations were measured during thermocycling and control conditions at infusion rates (IR) of 0.5, 1.0, 2.0, and 5.0 mL/h for both isoflurane and sevoflurane; an additional infusion rate of 10.0 mL/h was applied for sevoflurane. Before each measurement, the system remained undisturbed for at least 30 min to allow equilibration of gas concentrations; maximal fluctuations allowed were 0.05 Vol% (steady state). Anesthetic concentrations for isoflurane (C_ISO_) and sevoflurane (C_SEVO_) were then recorded over 5 min. Each measurement was repeated three times.

### Reflection efficiency

For both anesthetic agents, isoflurane and sevoflurane, reflection efficiencies (RE) were calculated as previously published [11], based on the measured lung concentrations of C_ISO_ and C_SEVO_:

$${\mathrm{RE}}_{{{\mathrm{ISO}}/{\mathrm{SEVO}}}} \left( \% \right) \, = { 1}00 \times \left[ {{1} - \left( {{1}00 \times {\mathrm{IR}} \times {\mathrm{F/}}\left( {{6}0 \times {\mathrm{C}}_{{{\mathrm{ISO}}/{\mathrm{SEVO}}}} \times {\mathrm{VT}} \times {\mathrm{RR}}} \right)} \right)} \right]$$

where IR is the infusion rate [mL/h], F is the factor for converting liquid anesthetic to vapour (isoflurane: 200 mL/mL, sevoflurane: 193 mL/mL), C_ISO/SEVO_ is the concentration isoflurane or sevoflurane [Vol%], VT is the tidal volume [mL], and RR is the respiratory rate [1/min].

### Data evaluation and statistics

Gas concentrations inside the test lung were recorded in steady state to two decimal places every 20 ms for 5 min, transferred to a computer using Visia™ software (Draeger Medical), and averaged. All measurements were repeated three times and again averaged. Anesthetic concentrations at different thermocycling levels were plotted against the infusion rates of both anesthetics and fitted using regression analysis. Repeated measures analysis of variance (RMANOVA) was performed with infusion rates and levels of thermocycling for isoflurane and sevoflurane separately. Neighbouring levels of thermocycling and control conditions were compared using paired Student’s *t* tests.

In addition, reflection efficiencies were plotted against the achieved concentrations for each thermocycling level.

RMANOVA and paired *t* tests were performed using IBM SPSS Statistics, Version 30 (IBM Corporation, Armonk, NY, USA). A *p* value < 0.05 was considered statistically significant. No corrections for multiple testing were applied.

## Results

**Fig. 2:**
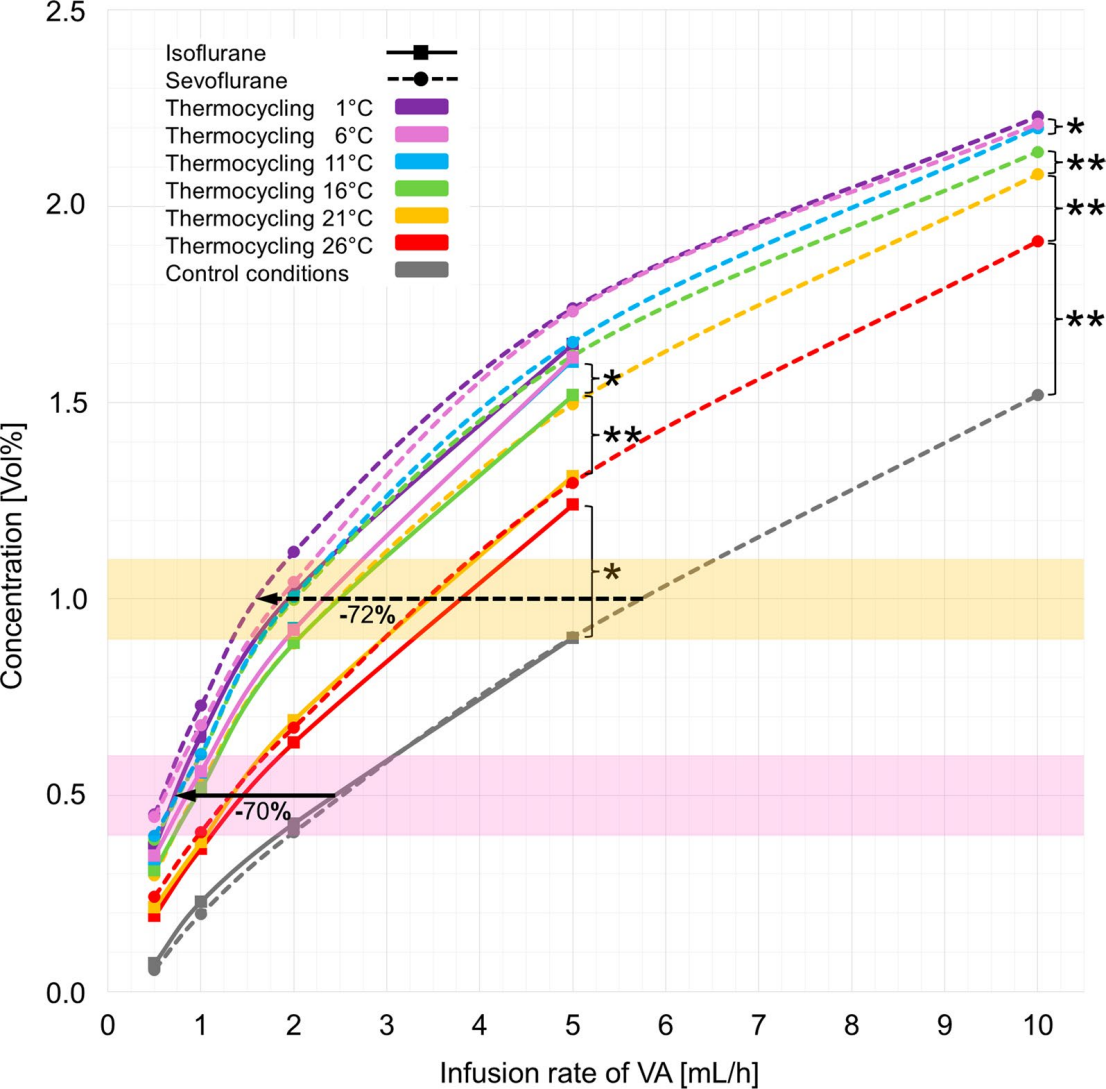
Anesthetic concentration [Vol%] at different infusion rates of volatile anesthetics (VA) [mL/h] under various thermocycling levels and control conditions. The markings indicate the concentration range regularly used in intensive care units (yellow: sevoflurane; violet: isoflurane). Significant differences between thermocycling levels: *p < 0.05; **p < 0.01, paired *t* test

The concentrations of both anesthetics increased as infusion rates rose, as well as with more intensive thermocycling (RMANOVA, isoflurane: *p* < 0.001 for infusion rates and thermocycling levels; sevoflurane: *p* < 0.001 for infusion rates and thermocycling levels). Reducing the temperature of exhaled air in 5 °C steps led to significant increases in concentrations at first, followed by progressively smaller rises. Statistically significant differences are marked in Fig. 2. Interpolation shows that for concentrations typically used for ICU sedation (isoflurane: 0.4–0.6 Vol% [1, 12]; sevoflurane 0.9–1.1 Vol%, [13]), infusion rates could be decreased by 70% and 72%, respectively (Fig. 2).

**Fig. 3:**
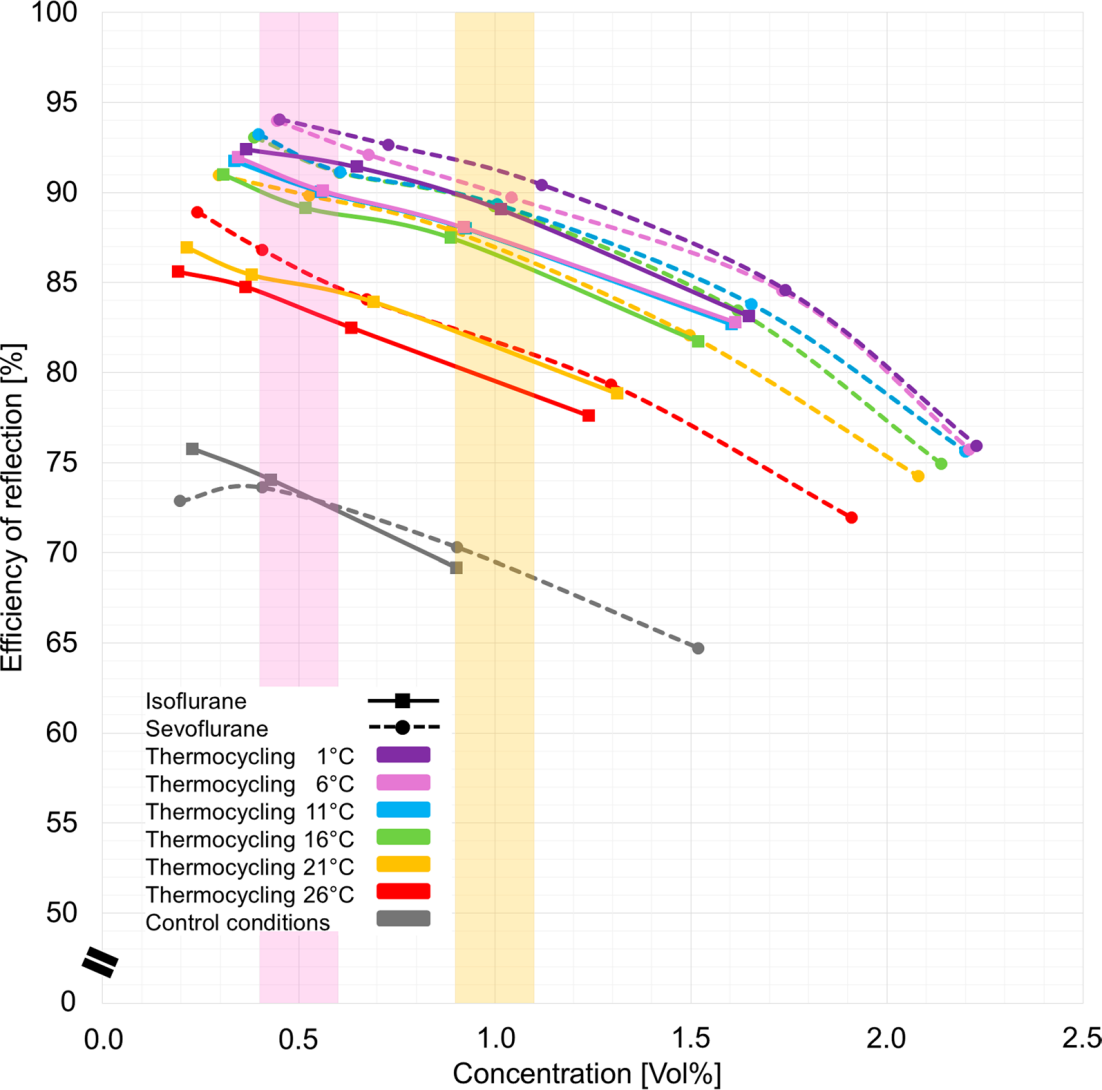
Reflection efficiency [%] vs. concentration of volatile anesthetics [Vol%] at different levels of thermocycling*.* The markings indicate the concentration range regularly used in intensive care units (yellow: sevoflurane; violet: isoflurane)

Thermocycling increased reflection efficiencies from around 70% under control conditions to around 90% for concentrations used for ICU sedation. Again, significant increases at first are followed by progressively smaller rises. Thermocycling with cooling temperatures of only 16 °C (green lines in Fig. 3) already shows considerable effects. As expected, reflection efficiencies decreased with higher concentrations, exceeding the capacity of the small ACD-S under evaluation (Fig. 3).

## Discussion

This study shows that the efficiency of isoflurane and sevoflurane reflection by the Sedaconda ACD-S can be considerably improved by thermocycling. Anesthetic reflection refers to the process of adsorption of the anesthetic by the carbon reflector inside the ACD during expiration and subsequent release back to the patient during the next inspiration. Thermocycling refers to cooling the reflector during expiration to facilitate anesthetic adsorption and heating it during the next inspiration to facilitate desorption of the anesthetic.

Heating during inspiration may simply and safely be achieved by heating up the inspired air to 37 °C. For this purpose, commercially available active humidifiers can be used. Heating alone has small to moderate effects on reflection efficiency, as previously shown by our group [14].

Cooling the reflector during expiration is more challenging. We chose to cool the expired air before it reaches the reflector. However, it must be avoided that subsequently cool and dry air is re-inspired to avoid patient harm. Therefore, a small breathing circuit was introduced between the ACD and the test lung with unidirectional valves. The expired air was directed through a copper tube passing through a freezer before reaching the ACD. With modifications of this expiratory limb, we could control the cooling temperature in steps of 5 °C from 26 °C down to 1 °C. Our results show substantial increases in the anesthetic concentrations inside the test lung in steady state down to a cooling temperature of 16 °C; below 16 °C, further increases in concentrations were much smaller.

Reflection efficiency refers to the proportion of re-inspired anesthetic molecules relative to the total number of molecules exhaled during a single breath. Previous laboratory studies reported an efficiency of approximately 90% for isoflurane and sevoflurane [8]. However, under BTPS and normocapnic conditions, reflection efficiency was observed to decrease to around 70% [9], which aligns with our control conditions. Our findings demonstrate that thermocycling can restore efficiency to levels exceeding 90%. This indicates that less than 10% of anesthetic is lost per breath, rather than 30%, resulting in a reduction of more than two-thirds (70% isoflurane; 72% sevoflurane) in anesthetic consumption.

Our previous study [10] demonstrated that thermocycling enhances isoflurane reflection. We used the same inspiratory hose heating setup and cooled the ACD-S by passing expired gas through ice. However, only BTPS conditions were applied, with no carbon dioxide, resulting in higher baseline reflection efficiency and a reduced net effect of thermocycling. This new study tested various thermocycling levels by lowering breathing gas temperature in 5 °C decrements to 1 °C, aiming for an optimal setting. The data suggest that an expiratory reflector temperature of about 16 °C is sufficient, as additional cooling did not improve reflection efficiency much further.

Reflection may also be improved by increasing carbon reflector mass, as in the older version Sedaconda ACD-L, but this also adds dead space, reducing usability for patients with small lungs or ARDS [9]. Thermocycling does not necessarily cause this issue. In our setup, extra breathing tubes between the ACD and test lung do not add dead space due to unidirectional valves blocking CO₂ rebreathing.

Using thermocycling in clinics may reduce drug costs, workplace concentrations, and workload by allowing longer intervals between anesthetic refills. Thermoelectric heat pumps using the Peltier effect could directly cool the reflector; applying direct current to these devices makes one side cold and the other hot, and reversing the current swaps the effect [15]. Therefore, heating during inspiration would be possible with one device and could also boost airway humidity, like active humidification.

Volatile anesthetics are potent greenhouse gases. In our laboratory setting, thermocycling cut their usage by 70% and 72%. While volatile anesthetic capturing systems (AGSS) with incineration or recycling are under investigation, reported capture rates are only 25% to 45% [16, 17]. Fabrication and transport of the capturing canisters, as well as combustion products, will add to the footprint of the anesthetics released into the atmosphere [18]. Due to the limitations of capturing and recycling technologies, increasing efficiency of volatile anesthetic reflectors thus holds great promise for reducing climate impact.

The Sedaconda ACD-S is primarily used for delivering inhaled sedation to critically ill patients. It can also decrease anesthetic consumption in the operating room, particularly during shorter procedures, by delivering quantitative boluses to rapidly raise anesthetic concentrations, rather than relying on high concentrations with increased flow rates for wash-in when using standard anesthesia machines [6]. A more sophisticated stand-alone reflection device, the Mirus™ (Technologie Institut Medizin GmbH, Koblenz, Germany), with anesthetic injections during inspiration, and target control of the anesthetic concentration, is commercially available [11]. Using bolus injections and anesthetic reflectors improved by thermocycling in anesthesia machines may significantly lower the carbon footprint of inhaled anesthesia in the future.

## Limitations

Implementing thermocycling in clinical settings will necessitate considerable engineering development. Miniaturization is required before potential clinical application, e.g., by direct cooling of the reflector through compact thermoelectric coolers or Peltier elements. Any adopted technical approach must ensure that only warm and humid air can be inspired by the patient. High reflection efficiency may *hinder reducing anesthetic concentration*, but this could be prevented by bypassing the reflector or simply by turning off thermocycling.

During the experiments, an HME was placed between the test lung and the additional breathing circuit to prevent humidity from entering the copper tubes inside the freezer. In a pilot study without HME, condensation and freezing resulted in ice formation that blocked the tubes. The use of this HME increases dead space and is generally not required, since the ACD includes HME functionality. In this experimental investigation using a test lung, standardized parameters for humidity, temperature, and ventilator settings were applied. Such standardization is rarely achieved in critically ill patients, which makes direct translation to intensive care unit conditions difficult. Nevertheless, this experimental setup demonstrates the increase achieved through thermocycling and thus represents a potential approach and contribution toward improving sustainability. Further studies involving potential patient application, as well as additional engineering expertise, are required to facilitate the transition from experimental research to clinical implementation.

An optimal benefit-to-effort effect for the use of thermocycling was demonstrated at 16 °C in this experimental study. Although a further increase in efficiency through more intensive thermocycling below 16 °C could be demonstrated, a progressively less favourable benefit-to-effort effect was observed in this lower range (Fig. 3). An efficiency much closer to 100% does not appear achievable with the present experimental setup and would likely only be attainable with a substantial expenditure of resources. Such an effort seems impractical for intensive care setting and, with view to the unfavourable benefit-to-effort effect, unlikely to be promising.

Separating the HME from the carbon reflector may be justified, as water vapour has been shown to reduce the reflection efficiency [9]. It is unclear whether this reduction is due to water competing with anesthetic molecules or due to a *reverse thermocycling effect*, in which water condensation raises the temperature and subsequent evaporation lowers it. In the Mirus™ device, the anesthetic reflector and HME are separated, with the HME positioned nearer to the patient [11].

We only evaluated a single ventilation setting (500 mL tidal volume, 12 breaths per minute) and selected infusion rates to maintain clinical anesthetic concentrations. In general, tidal volumes affect the resulting concentrations more than the respiratory rate, as higher tidal volumes will carry more anesthetic molecules to the reflector, occupying more binding sites, with excess molecules spilling over [8]. This will decrease reflection efficiency as shown in Fig. 3. Nonetheless, thermocycling still significantly improved it. The exact influence of tidal volume and respiratory rate is up to future studies.

## Conclusion

Thermocycling, i.e., cooling the reflector during expiration and heating it during inspiration, considerably improves the reflection efficiency of the Sedaconda reflection device. Our data suggest that an expiratory reflector temperature of about 16 °C is sufficient, as additional cooling showed much smaller effects. Miniaturizing the setup will be required before thermocycling can be used at the bedside.

## Data Availability

Data can be sent from the corresponding author upon reasonable request.

## Abbreviations

%: Percent
°C: Celsius
ACD: Anaesthetic Conserving Device
ARDS: Acute respiratory distress syndrome
BTPS: Body temperature pressure saturated
C_ISO_: Anesthetic concentrations for isoflurane
C_SEVO_: Anesthetic concentrations for sevoflurane
CO_2_: Carbon dioxide
e.g.: Exempli gratia, example
F: Factor for converting liquid anesthetic to vapour
HME: Heat Moisture Exchanger
i.e.: Id est, that is
ICU: Intensive Care Unit
IR: Infusion rate
L: Litre
L/min: Liters per minute
mL: Millilitre
mL/h: Milliliters per hour
min: Minutes
ms: Milliseconds
PEEP: Positive end-expiratory pressure
RE: Reflection efficiency
RMANOVA: Repeated measures analysis of variance
RR: Respiratory rate
T1: Thermometer
TM: Trademark
VA: Volatile anesthetic
Vol%: Volume percent
VT: Tidal volume
